# Exploring the effects of artificial intelligence on student and academic well-being in higher education: a mini-review

**DOI:** 10.3389/fpsyg.2025.1498132

**Published:** 2025-02-03

**Authors:** Blanka Klimova, Marcel Pikhart

**Affiliations:** ^1^Department of Applied Linguistics, Faculty of Informatics and Management, University of Hradec Kralove, Hradec Králové, Czechia; ^2^Centre for Basic and Applied Research, Faculty of Informatics and Management, University of Hradec Kralove, Hradec Králové, Czechia

**Keywords:** well-being, artificial intelligence (AI), higher education, quality of life, QoL divide artificial intelligence and AI

## Abstract

The increasing use of artificial intelligence (AI) in higher education is reshaping how students engage with their academic and personal lives. However, the impact of AI on students’ well-being remains underexplored. This mini-review synthesizes current literature to assess how AI affects student well-being, focusing on mental health, social interactions, and academic experiences. While AI offers benefits such as personalized learning, mental health support, and improved communication efficiency, it also raises concerns regarding digital fatigue, loneliness, technostress, and reduced face-to-face interactions. Over-reliance on AI may diminish interpersonal skills and emotional intelligence, leading to social isolation and anxiety. Furthermore, issues such as data privacy and job displacement emerge as AI technologies permeate educational environments. The review highlights the need for balanced AI integration that supports both academic success and student well-being, advocating for further empirical studies to comprehensively understand these dynamics. As AI becomes more embedded in education, it is crucial to develop strategies that mitigate its negative effects while promoting holistic well-being among students.

## Introduction

The rapid integration of artificial intelligence (AI) into higher education is reshaping how students engage with academic content and spend their free time, yet its impact on their well-being remains underexplored. Despite the growing use of AI in both academic tasks and personal activities, empirical studies on its effects on student well-being are notably scarce. This study addresses this gap by conducting a mini-review that seeks to synthesize the limited experimental and empirical evidence available on this critical issue. While the small number of studies reflects the early stages of research in this field, it is vital to establish a clear understanding of what is currently known. By doing so, this mini-review lays the groundwork for future empirical investigations, highlighting the importance of exploring how AI affects students’ mental health, social interactions, and overall well-being in higher education. Conducting this review is timely and necessary to create a foundation for further research, ensuring that the impact of AI on students is examined comprehensively as its use continues to expand.

In the context of higher education, AI-driven technologies are becoming indispensable tools for students, not only aiding their academic pursuits but also shaping the way they spend their leisure time (Chaudhary et al., 2024; Kamalov et al., 2023). From AI-powered learning platforms and personalized study assistants to entertainment apps and virtual communication tools, the role of AI in students’ lives has become more significant than ever (Bhutoria, 2022). Students spend increasing amounts of time in front of their screens, relying on AI for a variety of tasks, often at the recommendation of their instructors, who encourage them to leverage these technologies for assignments and research purposes (Seo et al., 2021). In many cases, students turn to AI to find quick answers, automate processes, or personalize their learning experiences (Kamalov et al., 2023). Outside of academia, AI-driven tools play an equally substantial role in entertainment, with students using these systems to engage in social media, gaming, and other forms of digital interaction during their free time (Dwivedi et al., 2023).

In addition, as research by Nakshine et al. (2022) maintain, excessive screen time can contribute to issues, such as digital fatigue, isolation, anxiety, and poor mental health. With the growing dependence on AI for learning, communication, and entertainment, students are exposed to digital environments that might lead to negative consequences for their overall well-being. The boundary between educational tasks and recreational activities is becoming increasingly porous, making it harder for students to manage their time effectively or engage in meaningful offline interactions (Dwivedi et al., 2023). This continuous engagement with AI technologies may, in turn, affect students’ ability to form social connections, relax without digital stimuli, or maintain a healthy balance between academic and personal life (Seo et al., 2021).

Given the centrality of AI in students’ educational and social lives, it is essential to evaluate its impact on their well-being critically. Understanding the broader implications of AI on students’ mental and emotional health is crucial not only for students themselves but also for educators and policymakers who are responsible for integrating AI technologies into curricula and student support systems (Shahzad et al., 2024). As AI-driven applications continue to evolve, assessing both the positive and negative aspects of their use is essential for creating informed strategies that promote not only academic success but also holistic student well-being (Chan and Hu, 2023).

This mini-review aims to explore the existing research on the impact of AI on students’ well-being in the higher education context. By synthesizing findings from various studies, this review provides a comprehensive overview of how AI-driven tools influence students’ mental, emotional, and social health, identifying key areas of concern and offering insights into potential strategies for mitigating the negative effects of AI usage. Understanding these dynamics is critical as higher education continues to embrace AI as a core component of learning, ensuring that student well-being is prioritized alongside academic innovation.

### Research questions

The research questions for this study were carefully developed to provide a clear overview of what is currently known about the impact of artificial intelligence on student well-being in higher education. Given the limited number of empirical studies available, these questions aim to synthesize the existing evidence, highlighting both the positive and negative effects of AI usage. By focusing on key areas such as mental health, social interactions, and communication skills, this review seeks to offer valuable insights that will serve as a foundation for further research and encourage future empirical studies in this evolving field.

1. How does the use of AI-driven educational tools influence students’ mental health and emotional well-being in higher education?
2. In what ways does AI usage, both for academic tasks and recreational purposes, impact students’ social interactions and communication skills?

These questions address the core aspects of well-being—mental health and social interaction—that are likely most affected by the integration of AI into students’ academic and personal lives.

## Methodology

This systematic review was conducted following the Preferred Reporting Items for Systematic Reviews and Meta-Analyses (PRISMA) guidelines to ensure a transparent and replicable process. To explore the impact of artificial intelligence (AI) on students’ well-being in higher education, two major academic databases were searched: Web of Science and Scopus. The inclusion criteria were strictly limited to experimental or empirical studies that investigated the effects of AI on student well-being within higher education settings. These types of studies, although limited in number, are crucial to establishing a reliable evidence base for understanding AI’s effects and guiding future research in this emerging field.

### Inclusion criteria

- Experimental or empirical studies that investigated the effects of AI on student well-being within higher education settings.
- Studies published in peer-reviewed journals.
- Studies available in English.

### Exclusion criteria

- Theoretical studies, reviews, or research that did not focus specifically on higher education or student well-being.
- Studies not available in full text.
- Studies not published in peer-reviewed journals.

The search string used in both databases was: *(“wellbeing” OR “well-being”) AND (“AI” OR “Artificial intelligence”) AND “higher education”*.

This search yielded a total of 180 studies from the Web of Science database. However, after applying the inclusion and exclusion criteria, only two studies were identified as relevant for this review. The Scopus search conducted for this study identified a total of 73 studies related to the impact of artificial intelligence on student well-being in higher education. However, after applying the inclusion and exclusion criteria, only 9 studies were found to be eligible for review. These studies met the criterion of being experimental or empirical research, which was essential for providing robust and reliable evidence. The review model deployed was a thematic analysis, which involved identifying, analyzing, and reporting patterns (themes) within the data. This approach helped in organizing and describing the data set in rich detail. Two experienced researchers independently reviewed the titles and abstracts of all identified studies. They manually selected the key studies based on the inclusion and exclusion criteria. Any discrepancies were resolved through discussion to ensure a consensus was reached.

Despite the small number of eligible studies, this mini-review aims to offer a comprehensive overview of the current knowledge on the topic and to highlight the need for further empirical research in this area.

The exclusion criteria included theoretical studies, reviews, or research that did not focus specifically on higher education or student well-being. Although the number of eligible studies was small, this review aims to provide a foundational analysis, paving the way for further empirical research on this important topic. This scarcity of experimental studies highlights the nascent stage of research in this area, underscoring the need for more empirical investigations to fully understand AI’s impact on student well-being in higher education contexts.

## Results

### Benefits of AI on student and academic well-being in higher education

One of the most significant benefits of AI in higher education is its ability to tailor educational experiences to individual students. AI-powered learning platforms use data-driven algorithms to assess student performance and adapt content to meet their specific learning needs (Zawacki-Richter et al., 2019). This personalized learning approach reduces stress by allowing students to progress at their own pace and receive targeted support, improving both academic performance and emotional well-being (Makhambetova et al., 2021). Shahzad et al. (2024) in their research conducted among 401 Chinese university students also indicate that students perceive AI as having the potential to positively influence mental well-being. They emphasize the potential of AI in strengthening mental health support systems because AI-powered chatbots and virtual assistants provide immediate support and essential information, making mental health services more accessible to a wider audience. This is in line with the findings of Labadze et al. (2023) who report that students benefit from AI-powered chatbots mainly in three key areas: assistance with homework and studying, personalized learning experiences, and skill development. Moreover, they also claim that for academics, it is especially the time-saving assistance and improved pedagogy. This has been recently supported by Cambra-Fierro et al. (2024) who conducted research among 401 university faculty members associated with the Business and Management fields. In particular, they discussed how the adoption of AI-driven educational tools, particularly ChatGPT, had influenced mental health and emotional well-being. They found out that ChatGPT adoption had increased overall happiness, reflecting a positive emotional impact. The use of AI reduced the stress associated with academic workloads, thereby contributing to higher happiness scores. In addition, increased energy levels were noted as a result of ChatGPT’s ability to simplify tasks, making academic work more manageable and improving student engagement and motivation. The findings also revealed that there had been a significant reduction in stress levels, as ChatGPT adoption alleviated the workload and provided immediate academic support, reducing the pressures of time management and task completion. Furthermore, the use of ChatGPT improved communication efficiency and collaboration in academic settings, especially in digital environments. Thus, AI helped students articulate ideas more effectively in written form, promoting clarity in academic exchanges. cAI-driven technologies can also facilitate broader access to educational resources for students with diverse needs. For example, AI tools can provide real-time language translation, assistive technologies for students with disabilities, and 24/7 access to tutoring and academic support (Luckin et al., 2016). These tools help create a more inclusive learning environment, reducing the anxiety and isolation that students with unique learning challenges often experience (Abbasi et al., 2024).

### Drawbacks of AI on student and academic well-being in higher education

On the contrary, research studies (Cambra-Fierro et al., 2024; Rodway and Schepman, 2023; Zhai et al., 2024) show that one of the key drawbacks in academic settings is the increasing reliance on AI tools. As Cambra-Fierro et al. (2024) state, over-reliance on AI for communication, especially in recreational contexts, may reduce face-to-face social interactions, negatively impacting interpersonal skills and emotional intelligence. Students may become more isolated and less adept at real-world social interactions and teamwork, which are critical to their overall social well-being and development. In addition, Crawford et al. (2024) in their study performed among 387 university students report that social support was found to mediate the relationship between loneliness and AI usage, suggesting that AI might increase loneliness when students perceive it as a primary source of support. As Rodway and Schepman (2023) expand, engaging with a technology that lacks empathy might be seen as an insufficient form of support, even though it has the capability to offer helpful advice. These findings were confirmed by Xie et al. (2023), who found a connection between increased AI use and loneliness but did not address the perception of AI as a social support system. Students who felt supported by AI experienced similar benefits in psychological well-being as those supported by humans, indicating that some students may turn to AI due to mental health issues or a lack of human support.

Generally, in higher education, students and academics may feel overwhelmed by the increasing reliance on AI tools, particularly when they lack sufficient training or technological literacy. This *technostress* (i.e., the anxiety and discomfort that individuals experience when interacting with new technologies) can negatively affect both mental health and academic performance (González-López et al., 2021).

Furthermore, the growing use of AI in higher education raises concerns about data privacy and security. AI systems often require access to vast amounts of student data, including academic performance, behavioral patterns, and even personal information (Akgun and Greenhow, 2022; Kamalov et al., 2023). This data is often used to enhance learning outcomes, but it can also lead to potential misuse or breaches of privacy. The fear of surveillance or loss of control over personal information can contribute to increased anxiety among students and staff, thus impacting their well-being (Malik et al., 2024).

Finally, the automation of various administrative and instructional tasks through AI raises concerns about job displacement for certain roles in higher education (Zawacki-Richter et al., 2019). Support staff, particularly those in administrative roles, may experience anxiety related to job insecurity as AI systems take over tasks such as student advising or scheduling. This professional uncertainty can negatively affect morale and lead to increased levels of stress (Budhwar et al., 2022; Erebak and Turgut, 2021).

## Discussion

Thus, on the findings described above, research shows that the key benefits of AI on student and academic well-being in higher education are as follows: personalized learning experiences (Zawacki-Richter et al., 2019), enhanced mental health support (Shahzad et al., 2024), time-saving for academics (Cambra-Fierro et al., 2024), inclusion of diverse learning needs (Luckin et al., 2016), and improved communication efficiency (Cambra-Fierro et al., 2024). On the other hand, the main drawbacks are reduced face-to-face interactions (Cambra-Fierro et al., 2024), increased loneliness (Crawford et al., 2024), technostress (González-López et al., 2021), data privacy and security concerns (Akgun and Greenhow, 2022), and job displacement concerns (Budhwar et al., 2022).

In practice, it means that educational institutions should consider balancing AI integration with human-centred approaches. While AI can enhance efficiency and personalize learning, over-reliance on it may compromise essential interpersonal skills and emotional intelligence. As Zhai et al. (2024) suggest, educational approaches should highlight the need to critically assess AI-generated content, compare it with insights from human sources, and recognize AI’s limitations. They also emphasize that it is essential to promote reflection on the inherent biases in AI outputs and involving students in tasks that require thoughtful analysis and integration of information from varied sources can cultivate a more informed and discerning use of AI tools Moreover, safeguarding data privacy should be a priority to mitigate the anxiety associated with surveillance. Institutions should also address the potential job displacement issues by upskilling staff to work alongside AI rather than be replaced by it since as Al-Zahrani (2024) points out, enhancements in AI transparency could simultaneously support teacher professional development and foster better student outcomes.

One of the primary limitations of this study is the novel state of research in the field, as the use of artificial intelligence in higher education and its impact on student well-being is still relatively new. As a result, there is a scarcity of experimental and empirical studies on the topic, which limits the depth and breadth of the analysis. Despite this limitation, the few studies available already demonstrate clear potential advantages and drawbacks of AI in relation to academic well-being. These findings provide an essential foundation for further research, highlighting both the benefits and concerns that need to be explored in more detail. Although the current body of literature is limited, it serves as a crucial starting point for more comprehensive, in-depth investigations into the long-term effects of AI on students’ mental health, social interactions, and overall well-being.

In summary, AI-driven educational tools influence students’ mental health and emotional well-being in higher education through both benefits and drawbacks. Positively, these tools offer personalized learning experiences, reduce stress by allowing students to progress at their own pace, and improve accessibility to mental health support via chatbots and virtual assistants. AI also promotes inclusivity by accommodating diverse needs, such as assistive technologies and 24/7 academic support. However, over-reliance on AI can lead to digital fatigue, technostress, and anxiety over data privacy concerns. While AI can enhance well-being by reducing academic pressures, its associated challenges require careful management.

Regarding social interactions and communication skills, AI use enhances digital communication efficiency and collaboration, particularly in academic settings. However, excessive reliance on AI for communication and recreational purposes can reduce face-to-face interactions, impairing interpersonal skills and emotional intelligence. It may also foster loneliness when AI becomes a substitute for genuine social support. The balance between leveraging AI for efficiency and maintaining meaningful human connections is essential to mitigate these negative impacts while maximizing its benefits.

As far as the practical or theoretical implications of the findings are concerned, educational institutions should adopt a balanced approach that combines AI’s efficiency with human-centred pedagogical methods to foster interpersonal skills and emotional intelligence. In addition, there is a need for policies addressing data privacy, ethical AI use, and training programs to improve technological literacy. Teachers should also incorporate critical thinking and AI literacy into curricula, which can help students evaluate AI-generated content and its limitations.

Future research into the impact of AI on academic well-being must be thus grounded in rigorous experimental and clinical studies to provide a more accurate understanding of what to expect as AI continues to play a dominant role in education. A serious academic discussion is required to address not only the direct effects of AI on student well-being but also the broader context of increased screen time, internet dependency, and potential AI addiction. As students spend more time engaging with AI-driven tools, both for academic tasks and recreation, it is essential to examine how this growing reliance affects their mental health, focus, stress levels, and academic performance. Without experimental data to guide our understanding, the long-term effects of AI on well-being remain speculative, making it critical to establish robust research frameworks that can produce actionable insights for educators and policymakers. Moreover, future studies must also address the intertwined issue of social isolation and the potential for addiction to AI technologies, particularly in conjunction with social media use. As AI systems increasingly personalize and optimize content, there is a growing risk that students may experience reduced face-to-face interactions and increased reliance on digital communication, which could exacerbate feelings of isolation. Additionally, research should investigate the ways AI-driven platforms contribute to addictive behaviors, such as compulsive screen use, which may further deteriorate students’ mental and emotional well-being. More work is also needed on ethical issues, such as data privacy, surveillance concerns, and their impact on students’ mental well-being. While the study discusses the benefits of AI for students with diverse needs, future research could focus on specific use cases and best practices to enhance its inclusivity.

Understanding these dynamics within the broader context of digital health will help educators develop strategies to mitigate the risks of over-reliance on AI while maximizing its educational benefits. By addressing these critical concerns through focused experimental and clinical research, future studies can offer clearer guidelines for integrating AI into educational systems in a way that prioritizes students’ well-being. This will ensure that as AI technology continues to evolve, its role in education is carefully managed to support—not undermine—the holistic development of students.
